# Limiting Excess Weight Gain in Healthy Pregnant Women: Importance of Energy Intakes, Physical Activity, and Adherence to Gestational Weight Gain Guidelines

**DOI:** 10.1155/2013/787032

**Published:** 2013-02-20

**Authors:** Tamara R. Cohen, Kristine G. Koski

**Affiliations:** School of Dietetics and Human Nutrition, Macdonald Campus, McGill University, 21,111 Lakeshore Road, Ste-Anne-de-Bellevue, QC, Canada H9X 3V9

## Abstract

Few studies have investigated if compliance with energy intakes, physical activity, and weight gain guidelines attenuate postpartum weight retention (PPWR) in mothers attending prenatal classes. We investigated whether (a) daily energy intakes within 300 kcal of estimated energy requirements (EERs), (b) walking more than 5000 steps/day, (c) targeting the recommended weight gain goals for prepregnancy BMI, and/or (d) achieving weekly or total gestational weight gain (GWG) recommendations minimized PPWR in 54 women attending prenatal classes in Montreal/Ottawa, Canada. Participants completed a validated pregnancy physical activity questionnaire (PPAQ), 3 telephone-validated 24-hr dietary recalls, and wore a pedometer for one week. PPWR was measured 6 weeks after delivery. Results showed that 72% had healthy prepregnancy BMIs. However, 52% consumed >300 kcal/day in excess of their EER, 54% exceeded recommended GWG, and more overweight (93%) than normal weight women (38%) cited nonrecommended GWG targets. Following delivery, 33% were classified as overweight, and 17% were obese. Multiple logistic regressions revealed that women targeting “recommended weight gain advice” were 3 times more likely to meet total GWG recommendations (OR: 3.2, *P* < 0.05); women who complied with weekly GWG goals minimized PPWR (OR: 4.2, *P* < 0.02). In conclusion, appropriate GWG targets, lower energy intakes, and physical activity should be emphasized in prenatal education programs.

## 1. Introduction

Research describes increasing rates of obesity in women of childbearing age [1, 2]. With more than 40% gaining in excess of Institute of Medicine recommendations [1], pregnancy is now emerging as an important risk factor for excessive weight gain [3] and an important target for obesity prevention studies [4]. Although most studies have focussed on multiethnic, socioeconomically disadvantaged obese women [1, 5–7], there is a growing concern that many healthy, university-educated, nonobese women may also gain excess weight during pregnancy, leading to postpartum weight retention (PPWR) and obesity later in life [3, 8]. In Canada, national statistics from the Maternity Experiences Study show that primiparous, university-educated women with medium- to high-household incomes gain more than is recommended when compared against recommendations released in 1999 [9, 10] or the newer revised 2010 guidelines [11, 12], with nearly 50% exceeding gestational weight gain (GWG) guidelines. The Canadian GWG guidelines [12] were harmonized with the US guidelines [13, 14] in 2010.

Despite the existence of GWG guidelines since the 1990's [10, 15], recent studies show that most women either receive no GWG advice [16–22] or target nonrecommended GWG goals [18, 23, 24]. Moreover, women no longer cite health professionals as their main source of information but identify the internet and family and friends as main sources of information [6, 24–27].

Because targeted goals [28], dietary and exercise interventions [29], and physical activity (PA) levels [30] have been associated with limiting excessive GWG, our objectives were (1) to investigate in healthy nonobese women attending prenatal classes in Ottawa/Montreal, Canada, if Health Canada's GWG targets [12] were being followed by our college-educated prenatal class attendees and (2) to identify specific behaviours that might be associated with achieving current Health Canada's GWG recommendations and/or minimizing postpartum weight retention (PPWR). We investigated the likelihood of achieving a healthy GWG and/or minimizing PPWR for the following four behaviours: (a) daily energy intakes within 300 kcal of estimated energy requirements (EERs); (b) walking more than 5000 steps/day; (c) targeting Health Canada's recommended GWG goals based on the mother's prepregnancy body mass index (BMI); and (d) achieving weekly or total GWG recommendations established by Health Canada [12] for healthy nonobese mothers.

## 2. Methods

### 2.1. Recruitment

 From 18 prenatal classes held in either Ottawa, Ontario, or Montreal, Quebec, between August and December 2008, 142 women were approached in their second and third trimesters to participate in this study. Ethics approvals were obtained from McGill University, Ottawa Public Health Ethics Board, and Centre de Santé de Services Sociaux (CSSS) Montreal (West Island and Cavendish boards). Researchers who were trained clinical nutritionists briefly described the study elements in a five-minute presentation at the beginning of each prenatal class, and interested clients provided their contact information at the end of class. In Canada, prenatal classes are voluntary and encouraged for first-time parents to learn about all aspects of pregnancy, delivery, and how to care for your newborn. These sessions are offered in both English and French and are free of charge. 

Signed consent was obtained for 81 mothers; 54 mothers completed all phases of the study that included a “GWG advice” questionnaire, 3 24-hr dietary recalls obtained by phone on nonconsecutive days, and wearing a pedometer for 7 consecutive days during the same week in which the dietary records were completed; 27 were excluded because they did not complete 3 dietary recalls or wear the pedometer for 7 consecutive days. Final inclusion criteria were for women >12 weeks of pregnancy, free of medical risks for PA, as described in the Physical Activity Readiness Medical Examination for Pregnancy (PARmed-X for Pregnancy) [31], and women who were not underweight (prepregnancy BMI < 18 kg/m^2^) or obese (prepregnancy BMI > 30 kg/m^2^) and did not have a multiple pregnancy, which is a contraindication as per the PARmed-X.

### 2.2. Provider Advice Questionnaire

 During the first visit, women were interviewed by the principle researcher, a licensed dietitian, on sources of GWG advice. Categories included physician and/or other health professionals, family and friends, and internet or books. The amount of weight each pregnant women was advised to gain was also recorded. Responses were categorized into the following weight ranges: <6.8 kg, 9.1–10.9 kg, 11.3–13.1 kg, 13.6–15.9 kg, >15.9 kg, or “no one discussed weight gain with me.” Responses reported as ranges (e.g., between 11.3 and 15.9 kg) were recorded as the mean of a weight range category. The “recommended advice” was calculated using each women's individual prepregnancy BMI and was compared to the following Health Canada and Institute of Medicine recommendations: BMI < 18.5: 12.5–18 kg; BMI = 18.5–24.9: 11.5–16 kg; BMI = 25.0–29.9: 7–11.5 kg; BMI ≥ 30: 5–9 kg [12, 13].

### 2.3. Weight Assessment

Self-reported pre-pregnancy weight and height were obtained at the first home visit as previously described [24]; actual body weight using a Tanita scale was also measured at the first home visit. Weekly GWG was calculated using current pregnancy weight minus prepregnancy weight (kg) divided by week of gestation minus twelve, as previously reported [24]. Self-reported pre-pregnancy weight was defined as weight at conception, and gestational age was based on date of last menstruation. Women were also telephoned at delivery and 6-week postpartum and were asked to report both their measured weight recorded by the physician at the time of delivery and their 6-wk postpartum weight recorded at this routine doctor's visit. This 6-wk time point has also been previously used to represent the maximal fat mass gained during pregnancy [32–34]. Specifically, this 6-wk postpartum measurement is considered a valid early indicator of adipose tissue accumulation during pregnancy because, at this time point, maternal weight is no longer influenced by changes in blood volume arising from pregnancy and/or planned weight loss. PPWR was calculated based on the difference between this 6-wk postpartum weight and the women's self-reported pre-pregnancy weight.

### 2.4. Physical Activity and Dietary Intake

During a home visit, mothers were instructed on how to wear the pedometer by a certified sports nutritionist. PA was assessed using a New Lifestyles Digi-Walker SW-200 [*Step Into Health, Plainfield, IL, USA*] pedometer for one week. Women also completed a PA logbook that included wear time, total steps/day and total time bathing, swimming or napping times, and how these compared with pedometer values. Values were compared to public health recommendations where less than 5000 steps/day is classified as a sedentary lifestyle [35]. 

Women also completed 3 nonconsecutive telephone dietary recalls during the week they wore the pedometer. Dietary interview kits and training were provided to assist women with estimating food portion sizes during the telephone recalls. The Canadian Nutrient File 2007 [36] and ESHA Research Food Processor (version 9.1) (Salem, OR, USA) were used to analyze food recalls for total energy (kcals) [37]. These were compared to the estimated energy requirements (EERs) (kcal/day) [37]. EER were calculated using the formula from the Dietary Reference Intake which estimates the EER based on age, PA level, height, weight, and the additional requirement associated with pregnancy. For all energy calculations, weights measured during the first home visit were used as previously described [24]. In Canada, in contrast to USA, no increased energy intakes are recommended for the first trimester, but an additional 340 kcal is recommended for second trimester and an additional 450 kcal for the third trimester [10, 12]; these later two values were added to the following equation: EER = 354 − (6.91 × age [y]) + PA ×  {(9.36 × weight [kg]) + (726 × height [m])} [38].

### 2.5. Statistical Analyses

Data was analyzed using Statistical Analysis Software [Version 9.2, 2002-2003, SAS Institute Inc, Cary, NC, USA]. Univariate logistic regressions were used to compute odds ratios (OR) for (a) achieving recommended total GWG and (b) carrying less than 4.5 kg (10 lbs) of additional weight 6-week postpartum for each of our 4 behaviors. Statistical significance was  *P* < 0.05. 

## 3. Results

### 3.1. Population Characteristics

**Figure 1:**
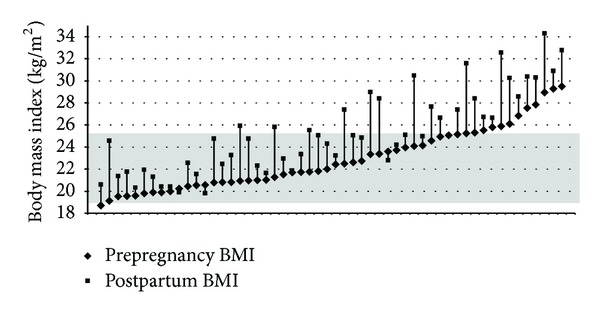
Comparison of Individual Mother's Pre-Pregnancy with their Post-Partum BMI. The differences in the individual weight gains for our 54 mothers are described. The shaded box represents the normal BMI category (18.8–24.9 kg/m^2^).

Table 1 describes our population characteristics. Participants were mostly Caucasian (85%), nulliparous (79%), and married (72%) women, with college (74%) or university degrees (26%) and household incomes >$50,000 (82%) who maintained “low active” lifestyles (6120 ± 2185 steps/day, range 840–11090 steps/day). Postpartum assessment revealed that the majority delivered healthy infants at term (39.3 ± 1.5 weeks) (3450 ± 494 g) either by vaginal delivery (68%) or planned caesarean section (32%). Energy intakes ranged from 1080 kg to 3760 kcal/day (mean 2240 ± 448); 54% exceeded their EER, individualized for their trimester, by more than 300 kcal per day. Total GWG ranged from 7.5 kg–35 kg (mean 17.3 kg). By 6 weeks postpartum, the majority of women with a pre-pregnancy BMI < 25 kg/m^2^ (54%) had retained >4.5 kg (mean 5.9 ± 4.9 kg), whereas 60% of overweight women (pre-pregnancy BMI = 25.0–29.9 kg/m^2^) had retained >4.5 kg (mean 8.2 ± 6.0 kg). Thus, although 72% began pregnancy having a normal pre-pregnancy BMI, based on their 6-week postpartum weight, 50% of study mothers were now classified as overweight or obese (*P* > 0.001) (Figure 1).

### 3.2. Information Sources

 The majority (76%) received advice about GWG: 49% from books/internet, 29% from physicians, 10% from other health care professionals (dietitian, nurse, and midwife), and 10% referenced all 3 sources. Twenty four percent of all study participants reported receiving no GWG advice. None cited their prenatal course as a source for their targeted GWG. Those who received practitioner advice or obtained information from books or the internet most often cited 13.6–15.9 kg as their targeted normal healthy GWG regardless of prepregnancy BMI. 

### 3.3. Adherence to “Recommended” GWG Targets

The majority (61%) of study participants followed incorrect GWG advice for their prepregnancy BMI. As well, 52% exceeded recommended weekly rates of GWG, normal prepregnancy BMI averaged 0.6 ± 0.2 kg/week; overweight pre-pregnancy BMI averaged 0.7 ± 0.3 kg/week. However, univariate logistic regressions showed that women who followed the correct total weight gain for their prepregnancy BMI were three times more likely to achieve Health Canada's GWG recommendations (OR: 3.2, *P* < 0.05) (Table 2). Women who achieved their recommended weekly rate of GWG were four times more likely to have retained less than 4.5 kg at 6-week postpartum (OR: 4.2, *P* < 0.02) (Table 2). Finally, women with a pre-pregnancy BMI < 25 kg/m^2^ were nine times more likely to target and to achieve Health Canada recommendations for GWG of 11.5–16 kg (Table 2). 

## 4. Discussion

Pregnancy is now considered obesogenic [3], but preventing excessive weight gain is proving to be a challenge [28–30]. In our study, we explored compliance with 3 measurable behavioural objectives and their impact on GWG and PPWR in women attending prenatal classes. GWG exceeded Health Canada recommendations in 52% of our study population, which is similar to other Canadian studies [9, 11, 39]. Our results showed that meeting GWG targets were associated with the following modifiable conditions and behaviours. First, a normal weight BMI < 25 kg/m^2^ increased the likelihood of mothers complying with Health Canada GWG guidelines, but a normal prepregnancy BMI did not prevent excessive PPWR, suggesting that behaviours associated with GWG and PPWR were not related to one another, which has been suggested [33]; secondly in both situations—for example, achieving an appropriate GWG or avoiding PPWR—neither energy intakes within 300 kcal of a mother's EER or becoming active and walking more than 5000 steps per day directly increased the likelihood of mothers achieving GWG recommendations or avoiding PPWR. Previous research has shown that purposeful walking, as measured by pedometer steps per day is related to weekly rate of GWG [24, 40], whereas higher energy intakes predict PPWR [33]. Thirdly our evidence strongly points to the important role for understanding and targeting recommended GWG guidelines and the importance of health care professionals conveying the message. It is well established that provider advice strongly impacts what expectant mothers actually gain [16, 22]. Should no advice or inappropriate weight gain advice be followed, previous studies show that women will exceed their GWG recommendations [9, 16, 22, 24]. Only 61% followed GWG guidelines for their prepregnancy BMI established by Health Canada. Others have reported lower rates [9, 11, 39].

Our GWG results were greater than a recent Australian study that reported that only 30% of normal weight women exceeded Institute of Medicine recommendations [41]. Similarly, recent Canadian studies identified 47% of primiparous mothers and 43% and 38% of college- and university-educated women, respectively, had excessive GWG; there were no differences for mothers from low (43%) versus high (41%) household incomes [9]. A second study reported nearly 50% exceeded GWG, but they found an association with income, ethnicity, and health status of the mother [11]. Taken together, these studies show that pregnant women attending prenatal classes who are not advised to meet GWG recommendations are at an increased risk of exceeding weight gain recommendations and of retaining in excess of 4.5 kg postpartum if they are sedentary throughout pregnancy as our study did. 

Strengths of our study include the fact that we measured energy intakes using 3 24-hr recalls and PA by both pedometers and a validated PPAQ [24]. Our previous modeling paper had identified interrelated causal pathways among energy intakes and PA and pregnancy outcomes that were not supported by direct relationships [33]. It is possible that increased PA and lower energy intakes were not associated with GWG and PPWR, which might be explained by the sedentary behavior of our mothers coupled with their high energy intakes. As well, pedometers are considered an acceptable method of assessing PA in pregnant women [42], their sensitivity to tilt angle may be more pronounced as pregnancy progresses and could affect recording of steps [43], thus underestimating PA.

Despite our small sample size, we believe our findings emphasize an important role for prenatal classes to educate pregnant women. Development of a public health promotion strategy for women attending prenatal classes should emphasize correct GWG based on the mother's prepregnancy BMI, appropriate energy intakes, and a nonsedentary lifestyle. In this study, university-educated women who received and followed the correct weight advice were more likely to avoid excessive GWG, and those who complied with weekly GWG which was achieved through “walking” [40], minimized PPWR, but none described their prenatal classes as having these objectives. This study demonstrates that by providing correct GWG targets in prenatal classes early in pregnancy more women can more easily achieve the Health Canada and Institute of Medicine recommendations for GWG and avoid PPWR.

## Figures and Tables

**Table 1:** Population characteristics (*n* = 54).

Characteristic	(mean (SD))
Age (y)	32.0 ± 4.3
Height (m)	1.7 ± 0.1
Gestational age (weeks)	26.8 ± 6.3
Pregnancy weight assessments	
Prepregnancy body mass index (kg/m^2^)^a^	23 ± 3
Estimated energy requirements (kcal/day)^b^	2341 ± 151
Average steps per day (steps/day)^c^	6133 ± 2203
Average energy expenditure (MET-hrs/day)^d^	6.6 ± 2.6
Total gestational weight gain (kg)	17.1 ± 6.4
Weekly gestational weight gain (kg)	0.71 ± 0.44
Postpartum weight assessments	
Weight retention at 6-wk postpartum (kg)^e^	10.9 ± 4.5
Postpartum body mass index (kg/m^2^)	25 ± 4

^a^Based on self-reported weight.

^b^Calculated using self-reported prepregnancy weight, self-reported height, age, and physical activity level.

^c^Steps per day determined by 7-day wear time of pedometer.

^d^MET-hrs/day determined by the pregnancy physical activity questionnaire [24].

^e^Weight retention at 6-wk postpartum was determined by subtracting self-reported pre-pregnancy weight from the 6-wk value measured at the time of their routine 6-wk postpartum doctor's visit.

**Table 2:** Odds ratios identifying behaviours associated with achieving a healthy GWG and minimizing PPWR in pregnant women (*n* = 54) attending prenatal classes.

	Odds ratio	95% Confidence interval	*P* value
Behaviours associated with achieving an appropriate total gestational weight gain (GWG)			

Normal prepregnancy BMI (18.5–24.9 kg/m^2^)	9.6	1.88–48.99	0.0065
Consuming within 300 kcal/day of EER	1.1	0.36–3.16	0.9005
Walking >5,000 steps/day	1.4	0.35–3.78	0.8271
Following “correct” total weight gain guidelines	3.2	1.04–9.85	0.0426

Behaviours associated with achieving <4.5 kg (10 lbs) postpartum weight retention (PPWR)			

Normal pre-pregnancy BMI (18.5–24.9 kg/m^2^)	1.3	0.38–4.31	0.6839
Consuming within 300 kcal/day of EER	1.3	0.45–3.84	0.6257
Walking >5,000 steps/day	1.1	0.34–3.74	0.8385
Following “correct” total weight gain guidelines	1.3	0.45–3.84	0.6257
Achieving Health Canada's recommended average weekly GWG rate (kg/week)	4.2	1.33–13.27	0.0147
